# Bayesian semi-parametric spatial modelling of intimate partner violence in Namibia using 2013 Demographic Health Survey Data

**DOI:** 10.1186/s12905-021-01421-2

**Published:** 2021-08-05

**Authors:** Oludoyinmola Ojifinni, Innocent Maposa, Latifat Ibisomi

**Affiliations:** 1 School of Public Health, University of the Witwatersrand, Johannesburg, South Africa; 2 Division of Epidemiology and Biostatistics, Wits School of Public Health, University of the Witwatersrand, Johannesburg, South Africa; 3 Health Science Research Office, Faculty of Health Sciences, University of the Witwatersrand, Johannesburg, South Africa; 4 Nigerian Institute of Medical Research, Lagos, Nigeria

**Keywords:** IPV risk, Spousal age difference, Non-linear effects in IPV, Spatial variation of IPV

## Abstract

**Background:**

Intimate partner violence (IPV) is an important public health problem with health and socioeconomic consequences and is endemic in Namibia. Studies assessing risk factors for IPV often use logistic and Poisson regression without geographical location information and spatial effects. We used a Bayesian spatial semi-parametric regression model to determine the risk factors for IPV in Namibia; assess the non-linear effects of age difference between partners and determine spatial effects in the different regions on IPV prevalence.

**Methods:**

We used the couples’ dataset of the 2013–2014 Namibia Demographic and Health Survey (DHS) obtained on request from Measure DHS. The DHS domestic violence module included 2226 women. We generated a binary variable measuring IPV from the questions “ever experienced physical, sexual or emotional violence?” Covariates included respondent’s educational level, age, couples’ age difference, place of residence and partner’s educational level. All estimation was done with the full Bayesian approach using R version 3.5.2 implementing the R2BayesX package.

**Results:**

IPV country prevalence was 33.3% (95% CI = 30.1–36.5%); Kavango had the highest [50.6% (95% CI = 41.2–60.1%)] and Oshana the lowest [11.5% (95% CI = 3.2–19.9%)] regional prevalence. IPV prevalence was highest among teenagers [60.8% (95% CI = 36.9–84.7%)]). The spatial semi-parametric model used for adjusted results controlled for regional spatial effects, respondent’s age, age difference, respondent’s years of education, residence, wealth, and education levels. Women with higher education were 50% less likely to experience IPV [aOR: 0.46, 95% CI = 0.23–0.87]. For non-linear effects, the risk of IPV was high for women ≥ 5 years older or ≥ 25 years younger than their partners. Younger and older women had higher risks of IPV than those between 25 and 45 years. For spatial variation of IPV prevalence, northern regions had low spatial effects while western regions had very high spatial effects.

**Conclusion:**

The prevalence of IPV among Namibia women was high especially among teenagers, with higher educational levels being protective. The risk of IPV was lower in rural than urban areas and higher with wide partner age differences. Interventions and policies for IPV prevention in Namibia are needed for couples with wide age differences as well as for younger women, women with lower educational attainment and in urban and western regions.

**Supplementary Information:**

The online version contains supplementary material available at 10.1186/s12905-021-01421-2.

## Background

Intimate partner violence (IPV) refers to all forms of behaviour within an intimate relationship that causes physical, sexual or psychological harm, including acts of physical aggression, sexual coercion, psychological abuse and controlling behaviours [1]. IPV has been recognized globally as an important public health problem [2] and has health and socioeconomic effects on women, children, men and entire communities [2–4]. Occurrence of IPV is particularly high in the countries of sub-Saharan Africa [2]. The global proportion of women reporting experience of IPV at least once in their lifetimes is a wide range from 6 to 59% [5]. In a multi-country survey of 81 countries the lifetime prevalence of physical and/or sexual IPV was 29.4% among ever-partnered girls aged 15–19 years and 31.6% among young women aged 20–24 years [2]. In sub-saharan Africa, the prevalence of IPV ranges from 57.6% in Cameroon, 53.9% in Zambia, 45.5% in Mozambique, 45.3% in Kenya, 43.4% in Zimbabwe and 30.5% in Nigeria [6].

Factors that have been associated with IPV include age and age asymmetry between partners, women’s education and employment status as well as societal norms [2]. The evidence from previous research on the association between age and experience of IPV appears contradictory. While one multi-country study found women at both extremes of age had a high risk of experiencing IPV [7], a meta-analysis examining risk markers for IPV found older age to be a protective factor against experiencing IPV among women [8]. The evidence on the relationship between education and experience of IPV also appears contradictory. Some studies have reported a hump-shaped or a reverse “U” shaped distribution of IPV in association with women’s level of education where women at the extremes have lower risk of experiencing abuse than those in the middle [9–11]. Other studies show lower levels of IPV among women who have secondary education or higher [2, 7, 12] while some others show no relationship between education and IPV [13, 14].

Societal norms that influence the experiences of IPV include cultural ideals that condone the subjugation of women, women’s acceptance and justification of IPV, and the acceptance of IPV as part of daily life [2, 15–17]. In many sub-Saharan African countries where there is acceptance of abuse as normative, the risk of IPV is reportedly higher for women with higher educational and economic status [10]. With respect to women’s employment status some studies show increase in experience of IPV while others show a reduction among women who were employed depending on the geo-cultural context [2]. In areas where there is societal acceptance of domestic violence or high levels of violence in the community, female employment is associated with higher risk of IPV [10]. Furthermore, an attempt to exact dominance on women seen to be gaining independence through employment or to extract resources from the women can lead to a “violence backlash” that increases their risk for experiencing IPV [10]. On the other hand, having a high household socioeconomic status is protective against IPV [10, 18].

Domestic violence is recognized as an endemic problem in Namibia affecting both sexes and all ages [19]. The lifetime prevalence of IPV in a hospital-based study in Namibia was 10.1% with the most common form being emotional abuse [13]. A review of existing literature documented that 16.5% of Namibian women aged 15–49 years had experienced sexual coercion by an intimate partner [20]. Findings from the 2013 Namibia Demographic and Health Survey showed that 33% of ever-married women aged 15–49 years had ever experienced any form of domestic violence with 28% being in the 12 months prior to the survey [19]. The proportion of ever-married women who had ever experienced physical violence at least once since age 15 was 32% of whom 50% was perpetrated by their current partners. Among those who experienced violence perpetrated by their current partners, 32% sustained physical injuries as a result of the violent acts [19]. Being unemployed, increased number of children, living in rural areas and having multiple sexual partners were associated with reporting experience of IPV among women in Namibia [19, 21]. A qualitative study among men who had been imprisoned for homicide resulting from IPV in Namibia found that many blamed the victims for being insubordinate or unfaithful [22].

Many studies that have assessed risk factors for domestic violence in general and IPV in particular have relied mainly on logistic regression and sometimes Poisson regression without incorporating the geographical location information and effects. Spatial statistics has grown rapidly in the past decade to explain the spatial effects associated with different outcomes in many fields especially epidemiology and public health [23]. In this study we leverage the strengths of generalized linear mixed effects models and the flexible parameter estimation in semi-parametric regression or additive models’ literature with Bayesian procedures to determine the risk factors for IPV in Namibia using the first nationally representative survey on IPV in the country. With this Bayesian spatial semi-parametric regression model, we will be able to assess the non-linear effects of age difference between partners as well as determine if there are spatially correlated effects in the different regions on the prevalence of IPV in Namibia.

## Methods

### Study area

Namibia is a middle-income country located in the southwestern region of Africa. Its borders are the Atlantic Ocean on the west, Angola and Zambia on the north, Botswana on the east and South Africa on the south and east. The country is divided administratively into 13 regions with the capital at Windhoek in the Khomas region. Namibia’s population was about 2.1 million from the 2011 census with an intercensal growth rate of 1.5% [19]. The country’s economy relies on agriculture, tourism and mining; although there has been rapid urbanization, the population is mostly rural with about four in ten people living in rural areas [19].

### Source of data and sample

Data used for this research was from the 2013–2014 Demographic and Health Survey (DHS) in Namibia. The request to use the data set was made to and permission obtained from Measure DHS. The DHS is a nationally representative cross-sectional survey which among other things monitors domestic violence and IPV, malaria, HIV, maternal and child health conditions as well as reproductive health issues. The DHS domestic violence module, from which our data is derived, used a shortened and modified conflict tactics scale (CTS) [24] to measure different forms of IPV [25] and domestic violence in general. The domestic violence questionnaire was administered, for the first time in the Namibia Demographic Health Survey (NDHS) 2013 [19], to a nationally representative sample of women between 15 and 49 years. In total 2226 women consented and responded to the domestic violence survey questions. For our study, we used the variables specific to spousal violence. We generated a new binary variable, which measures IPV in three dimensions from the questions: 1. Ever experienced physical violence? 2. Ever experienced sexual violence? and 3. Ever experienced emotional violence? Background characteristic variables such as region, place of residence, age, respondent’s level of education, partner’s educational level and wealth index level were considered as covariates. In addition, we generated another variable, age difference, from the respective ages of partners/couples in the dataset. We used the couples’ dataset for our analysis in this study. Since IPV may be associated with location of residence, it is important to account for geographical and cultural differences. We used region level effects to allow expected spatial correlation and any other unknown regional heterogeneity of IPV [26].

#### Statistical model

Let $y_{ij}$ be the intimate partner violence (IPV) status for a woman $i$ in region $j$. $y_{ij} = 1$ if the woman $i$ in region $j$ experienced some form of partner violence and $y_{ij} = 0$ otherwise. A vector $X_{ij} = (x_{ij1} ,x_{ij2} , \ldots ,x_{ijp} )^{{{{\prime}}}}$ contains $p$ continuous covariate random variables and $Z_{ij} = (z_{ij1} ,z_{ij2} , \ldots ,z_{ijr} )^{\prime}$ contains some r categorical variables. In our study, $p = 3$ and $r = 5$.

This study assumes that the dependent variable, $y_{ij}$ is a Bernoulli distributed random variable with $y_{ij} |p_{ij} \sim Bernoulli\left( {p_{ij} } \right)$ with an unknown $E\left( {y_{ij} } \right) = p_{ij}$, being related to the covariates through the link function

1 $$g\left( {p_{ij} } \right) = X_{ij}^{\prime} \beta + Z_{ij}^{\prime} \theta$$

The link function in this equation is known as the logit link, $\beta$ is the $p$ dimensional vector of coefficients for the continuous random variables, and $\theta$ is an $r$ dimensional vector of coefficients for categorical random variables. In order to assess for both non-linear effects of continuous random variables and spatial autocorrelation in our data we employed a semi-parametric model which utilizes a penalized regression approach [23]. The penalized regression approach is a non-parametric method of ordinary least squares (OLS) which relaxes the highly restrictive linear predictor for a versatile semi-parametric predictor [23, 27]. The flexible semi-parametric predictor is defined by:

2 $$g\left( {p_{ij} } \right) = \mathop \sum \limits_{v = 1}^{p} f_{v} \left( {x_{ijv} } \right) + f_{spat} \left( {s_{j} } \right) + Z_{ij}^{\prime} \theta$$

where $f_{v} \left( . \right)$ represents the non-linear twice differentiable smooth function for the continuous covariates and $f_{spat} \left( {s_{j} } \right)$ is the variable that denotes the spatial effects for each region. In our study, as in Ngesa et al. [23], we consider a convolution approach to the spatial effects. The assumption is that the spatial effects can be decomposed into two pure components, that is, spatially structured and spatially unstructured effects given as $f_{spat} \left( {s_{j} } \right) = f_{str} \left( {s_{j} } \right) + f_{{unstr\left( {s_{j} } \right)}}$. The final model for our study then becomes:

3 $$g\left( {p_{ij} } \right) = \mathop \sum \limits_{v = 1}^{p} f_{v} \left( {x_{ijv} } \right) + f_{str} \left( {s_{j} } \right) + f_{unstr} \left( {s_{j} } \right) + Z_{ij}^{\prime} \theta$$

More details on the model formulation are available in Additional file 1: Appendix 1.

## Data analysis and results

The weighted IPV prevalence was computed for all the 13 regions in Namibia as well as for other demographic characteristics among women. Unadjusted odds ratios were also computed for each of the demographic and women characteristics in the DHS dataset. For the final model, we assessed two plausible specifications, that is, a model with random effects only and the other with spatial and random effects in addition to covariates of interest. The BICs for the two models were 1648.07 and 1646.37 respectively, thus we selected the model that had spatial and random effects. The spatial autocorrelation assessment (Moran Index = 0.326, *P* = 0.01) was also significant indicating that regions close to each other are similar with regards to intimate partner violence.

### Observed risk of intimate partner violence in Namibia

As shown in Table 1, the country prevalence for IPV was 33.3% (30.1–36.5%). The highest regional prevalence of 50.6% (41.2–60.1%) was recorded in Kavango while the prevalence of 11.5% (3.2–19.9%) in Oshana was the lowest in the country. From the unadjusted odds, the probability of experiencing IPV was between three to eight times higher in Oshikoto, Hardap, !Karas, Erongo, Khomas, Omaheke, Zambezi, Otjozonjupa and Kavango than in Oshana. With respect to age, being 20 years and older appeared to be protective against IPV and the prevalence was highest among teenagers (60.8% [36.9–84.7%]). Living in either urban or rural locations made no significant difference to the experience of IPV as the prevalence was similar in both locations. Being of higher educational status was significantly protective [ORs: 0.52 (0.31–0.86); 0.39 (0.18–0.85)] for secondary and higher education levels compared with no formal education, respectively. In this study the prevalence of IPV reduced with increasing level of education from 47.1% (35.3–58.9) among those with no formal education to 26.1% (14.2–38.0%) among those with higher than secondary education. The prevalence of IPV reduced minimally with increase in partner’s level of education from 36.1% (26.5–45.6%) among women whose partners had no formal education to 34.5% (30.1–38.9%) among those whose partners had secondary education. The lowest prevalence 19.7% (11.5–27.9) was among women whose partners had higher than secondary education. Wealth status however was not significantly associated with the experience of IPV.

**Table 1:** Intimate partner violence prevalence and univariate logistic regression odds ratios for region, age group, residence, socioeconomic status and educational level for respondent

Variable		IPV prevalence			Weighted unadjusted odds ratios		
Region	N	n	Weighted %	95% CI	ORs	95% CI	*p* values
Whole country	1447	497	33.3	30.1–36.5			
Oshana	77	11	11.5	3.2–19.9	Ref		
Omusati	61	12	17.1	6.4–27.8	1.65	0.54–5.02	0.376
Ohangwena	67	17	19.5	9.3–29.8	1.93	0.67–5.57	0.221
Kunene	117	30	24.8	15.1–34.5	2.60	0.98–6.84	0.054
Oshikoto	80	34	43.2	30.3–56.2	6.04	2.26–16.17	< 0.001*
Hardap	111	35	31.8	22.1–41.5	3.58	1.43–8.96	0.006*
!Karas	113	42	37.9	27.4–48.3	4.67	1.87–11.66	0.001*
Erongo	160	46	27.9	19.9–35.9	2.93	1.20–7.14	0.018*
Khomas	134	47	34.4	24.3–44.4	3.95	1.59–9.82	0.003*
Omaheke	121	47	38.3	27.4–49.2	4.88	1.91–12.50	0.001*
Zambezi	131	48	36.2	27.1–45.2	4.46	1.79–11.11	0.001*
Otjozonjupa	126	51	42.9	32.5–53.3	5.81	2.33–14.45	< 0.001*
Kavango	149	77	50.6	41.2–60.1	8.13	3.27–20.23	< 0.001*
*Residence*							
Urban	763	260	32.8	28.3–37.3	Ref		
Rural	684	237	33.9	29.6–38.3	1.05	0.79–1.39	0.732
*Age group in years*							
15–19	33	17	60.8	36.9–84.7	Ref		
20–24	169	61	33.4	24.2–42.6	0.32	0.12–0.90	0.031*
25–29	268	86	31.5	24.3–38.7	0.30	0.11–0.80	0.017*
30–34	297	101	33.1	26.2–39.9	0.32	0.12–0.86	0.023*
35–39	267	95	33.3	25.9–40.7	0.32	0.12–0.87	0.026*
40–44	235	76	32.3	23.8–40.7	0.31	0.11–0.85	0.023*
45–49	178	61	32.1	23.4–40.7	0.30	0.11–0.84	0.022*
*Educational level*							
No formal education	122	50	47.1	35.3–58.9	Ref		
Primary	349	137	37.5	31.1–43.9	0.67	0.39–1.16	0.152
Secondary	854	282	31.6	27.6–35.6	0.52	0.31–0.86	0.010*
Higher	122	28	26.1	14.2–38.0	0.39	0.18–0.85	0.018*
*Partner’s educational level*							
No formal education	189	64	36.1	26.5–45.6	Ref		
Primary	274	106	34.9	27.3–42.6	1.04	0.59–1.81	0.902
Secondary	775	260	34.5	30.1–38.9	0.98	0.60–1.60	0.931
Higher	148	38	19.7	11.5–27.9	0.44	0.22–0.88	0.020*
Don’t know	58	28	42.3	27.0–57.5	1.65	0.76–3.59	0.203
*Wealth index level*							
Poorest	257	99	37.2	30.2–44.2	Ref		
Poorer	272	93	31.8	24.5–39.2	0.79	0.50–1.24	0.300
Middle	304	105	30.0	23.4–36.6	0.72	0.47–1.11	0.142
Richer	283	107	40.1	32.8–47.4	1.13	0.74–1.73	0.570
Richest	331	93	29.0	21.8–36.2	0.69	0.44–1.09	0.111

*Implies statistical significance i.e. *p* < 0.05

**Fig. 1:**
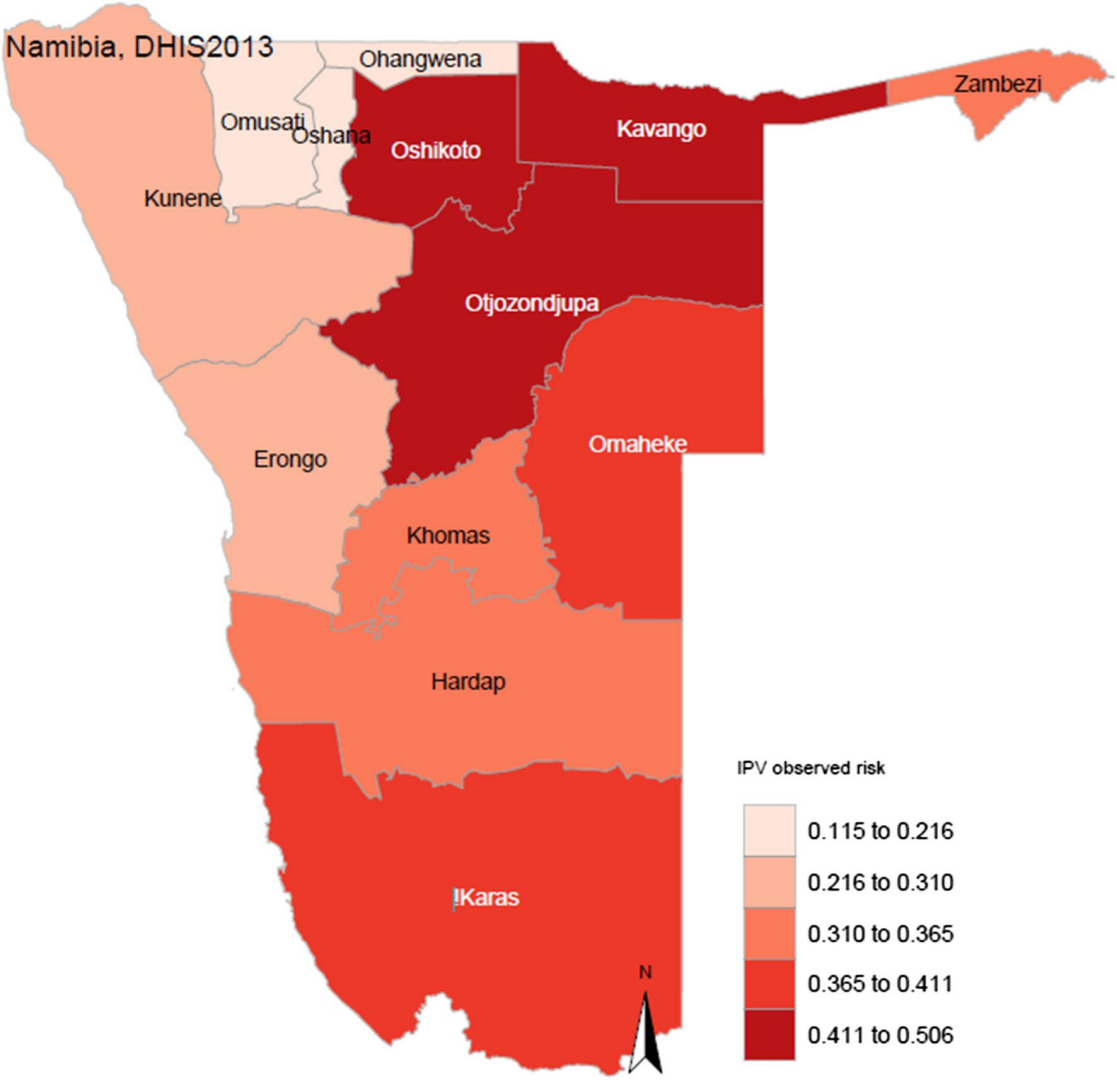
The observed IPV risk in ever married women in the 13 regions of Namibia (*This map was generated in the statistical software R using computed weighted regional prevalences of IPV and publicly available shape files for Namibia*)

Figure 1 shows the IPV prevalence in Namibia’s 13 regions. This figure shows that the North Eastern regions have higher risk of IPV than the other regions. Kavango region had the highest prevalence of IPV.

Table 2 shows adjusted odds ratios (aOR) and their corresponding 95% credible intervals (CIs) for categorical and fixed covariates. The risk of experiencing IPV was not significantly associated with place of residence after adjusting for regional spatial effects, respondent’s age, age difference, respondent’s years of education, wealth, and education levels. Women with higher education were significantly 54% less likely to experience IPV [aOR: 0.46, 95% CI 0.23–0.87]. No association was observed between wealth index level and place of residence with the probability of experiencing IPV.

**Table 2:** Parameter estimates and 95% credible intervals for the fixed effects

Explanatory variables	Adjusted OR	95% CI^1^ for aOR
*Residence*		
Urban (Ref)	1.00	
Rural	0.76	0.546–1.046
*Education level*		
Primary (Ref)	1.00	
Secondary	0.73	0.505–1.066
Higher	0.46	0.230–0.871*
*Wealth index level*		
Poorest	Ref	
Poorer	0.85	0.541–1.356
Middle	0.92	0.582–1.463
Richer	1.13	0.689–1.896
Richest	0.74	0.410–1.343
Respondent age	*	
Respondent years of education	*	
Age difference	*	

*CI*^*1*^ credible intervals

**Fig. 2:**
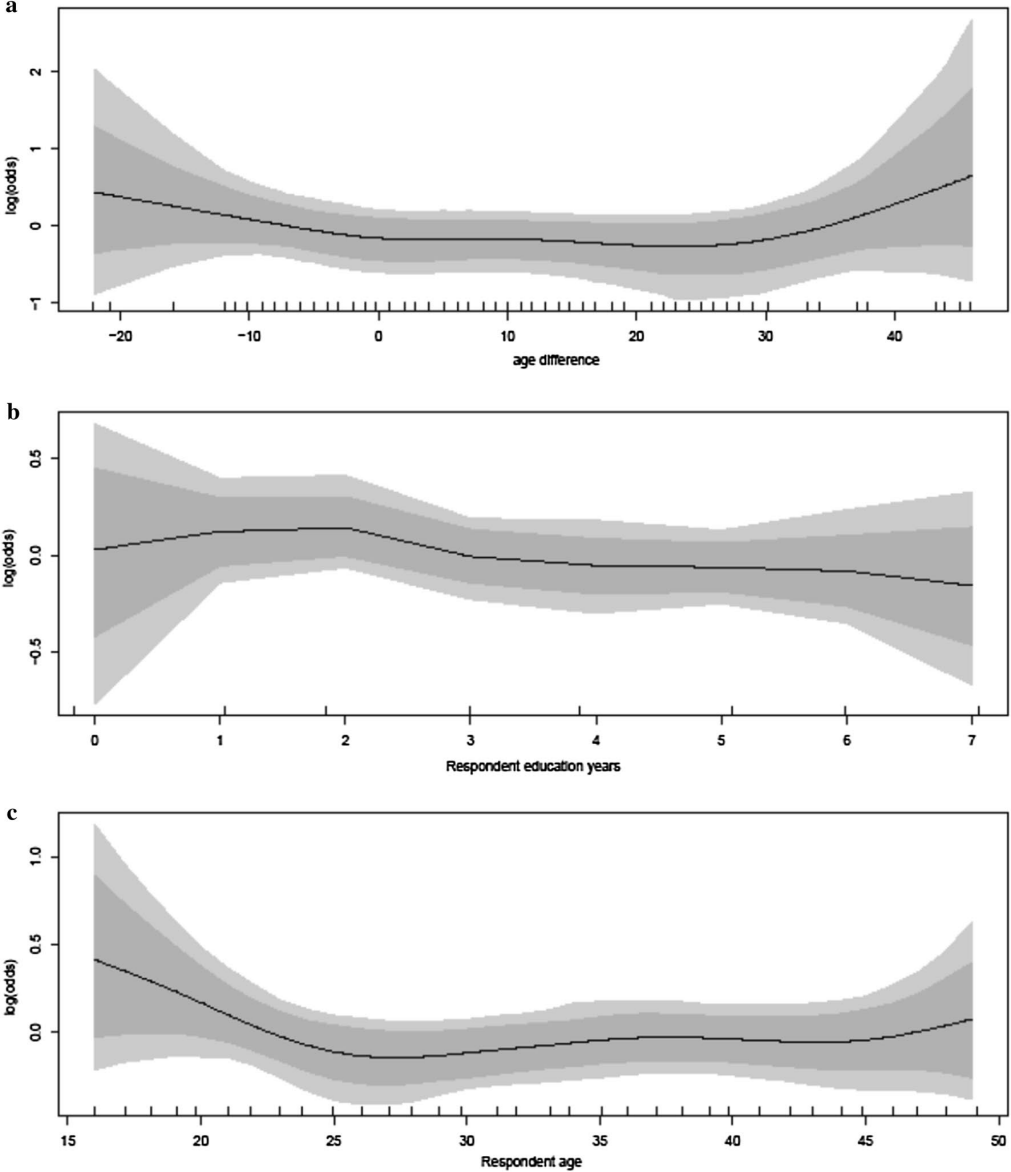
Effects of age difference (**a**), years of education (**b**) and woman's age (**c**) on the risk of experiencing IPV

*Non-linear effects (Figure 2a–c)

### Effects of continuous covariates on intimate partner violence

Figure 2 shows the nonlinear effects of women’s current age, number of years of education and age difference to partner on risk of experiencing IPV controlling for other variables. Figure 2a shows that the risk of experiencing IPV, in terms of the log odds ratio, is high for women who are roughly 5 or more years older than their partners and for women whose partners are at least 25 years older. Women with more years of schooling had lower probability of IPV (Fig. 2b). The pattern for risk of experiencing IPV with age is almost similar to that of age difference with younger and older women experiencing elevated risks compared to those between 25 and 45 years. The figure shows that the effects of these covariates are overall not linear on the likelihood of women experiencing IPV as the effects on the extremes of these covariates are different from the central. The assumption of linear effects for these variables on the risk of experiencing IPV would have led us to miss these subtle nuances which may be critical in proposing policies around girl child marriages and education. The confidence intervals for the non-linear effects for the three continuous covariates include zero indicating non-significant effects of these variables regardless of the observed patterns.

### Regional spatial and unexplained effects on IPV risk (log odds) map

**Fig. 3:**
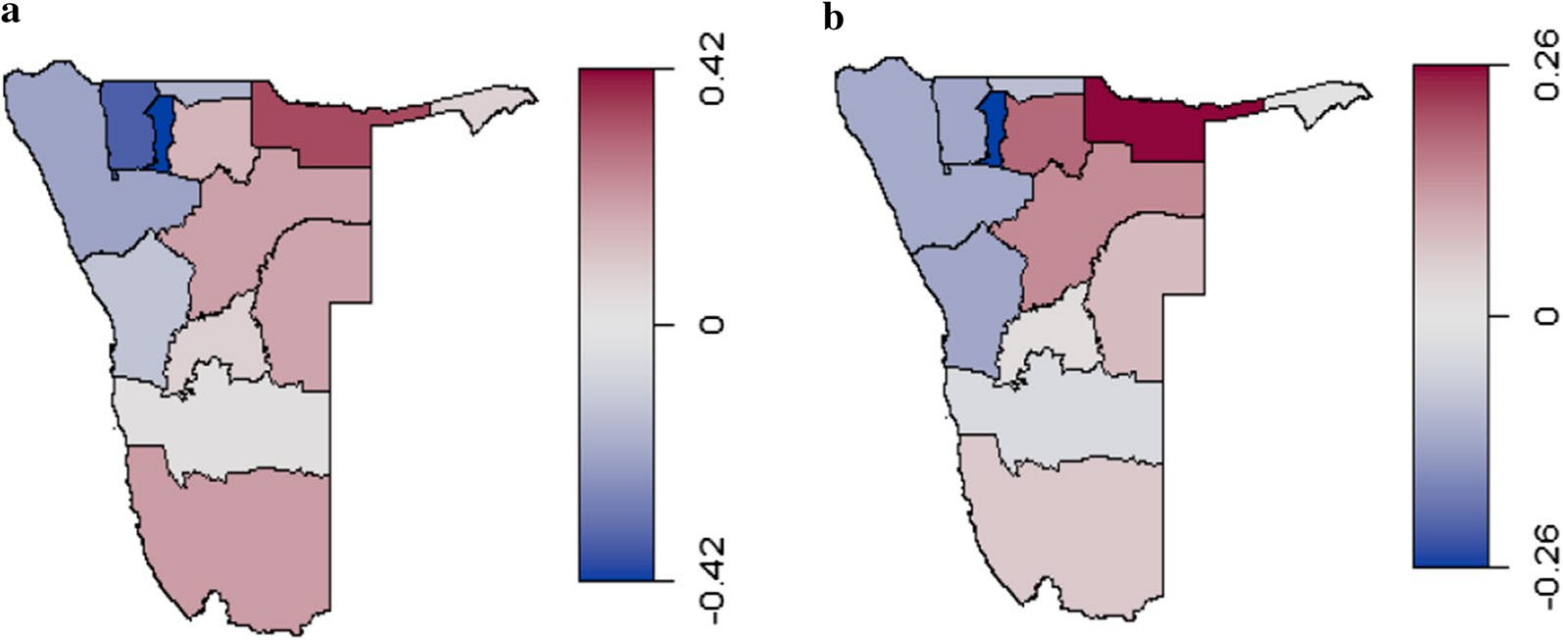
The regional spatial effects risk map for IPV in Namibia (**a**) and unexplained effects (**b**) on the risk of experiencing IPV for married women. (*These maps were generated in the statistical software R using computed weighted regional prevalences of IPV and publicly available shape files for Namibia*)

Figure 3 shows the IPV risk map for Namibia and the unexplained effects after controlling for respondent’s age, age difference, respondent’s years of education, wealth, and education level. Figure 3a shows that in general, regions in the Northern parts of Namibia show low association with IPV (negative log odds ratio) while north-eastern regions have high association (positive log odds ratio) with the occurrence of IPV. There is discernible evidence of spatial variation of IPV prevalence. From the maps, regions in the northern part of Namibia that include Oshana, Ohangwena, Omusati and Kunene show low IPV prevalence. The north-eastern parts of Namibia include regions like Kavango, Oshikoto and Zambezi which have high headcount poverty rates compared to other regions in Namibia show high IPV prevalence. Figure 3b shows a map of unexplained effects (residual effects) indicating higher effects in the same regions where higher regional spatial risk of IPV were noted.

## Discussion

This study assesses the risk factors for IPV in Namibia using spatial statistics to analyse the data from the first nationally representative IPV survey in the country. The unadjusted odds showed that older ages, higher educational attainment among women and their partners are protective against experience of IPV while wealth status was not significantly associated. On the other hand, spatial adjustment data showed lower risk of IPV in rural areas following adjustment for educational and wealth levels and region of residence. In addition, women with higher educational attainment had less likelihood of IPV after controlling for other variables.

The findings in this study with respect to educational attainment is consistent with the literature that shows lower risk of IPV among women who have secondary education or higher [2, 7, 12]. Similar to this study, a systematic review of studies in Ethiopia [28] and a cross-sectional study on determinants of IPV in Ghana using DHS data [29] both show that having secondary education or higher is protective against IPV. Our findings thus contrast with the inverted “U” as reported in an analysis of the Demographic and Health Surveys of 30 sub-Saharan African countries over the 10-year period of 2003–2013 [10]. This study showed that women with elementary and secondary education were more likely to experience abuse than those who had no formal education whereas women who had tertiary education had a lower risk of experiencing abuse [10].

With regards to partners’ educational attainment, the findings from this study is similar to that of the Ethiopian systematic review and the Ghanaian DHS data review where higher level of partner’s education is protective against IPV [28, 29]. Cools and Kotsadam [10] also showed that the risk of IPV is reduced when the partner has post-secondary education although this protection is lost when there is inequality in the educational status of the partners. Having fewer or more years of education than one’s partner is associated with increased risk of IPV [10]. Vyas and Watts on the other hand found from a review of literature that the risk for IPV increased when the woman had a higher level of education than her spouse [18]. They also reported that having secondary education was protective both for women experiencing and men perpetrating violence [18]. An analysis of the Nigerian 2013 DHS data also showed that increasing differences between the educational attainments of spouses predisposes to IPV risk [30]. The effect of differences in educational attainment may be due to a phenomenon described as status inconsistency [10, 31] where atypical roles in a relationship threatens a man’s identity and predisposes to violence. Women who have more resources—either due to higher economic power or higher education—are thus at increased risk of IPV [10].

Findings from literature reveal varying evidence with respect to lower risk of IPV in rural than urban areas. For instance, findings from the Ghanaian study show that rural dwellers had lower risk than those in urban areas [29]. In contrast, findings in Ethiopia revealed that the risk of IPV was higher in rural areas [28]. In our study, women living in rural areas were found to have lower risk of IPV when adjustment was made for educational and wealth level and region of residence, although this was not statistically significant. However, according to the Namibia 2013 DHS report, 37% of women in rural areas justify a husband hitting or beating his wife for any reason compared with 21.5% of women in urban areas. Also 12.6% of women in rural areas who worked for cash have their income solely controlled by their partners compared with 7% of women in urban areas. In addition, 7.7% of women in rural areas compared with 5.2% of women in urban areas do not make decisions concerning their own health care, major purchases in the family or visits to their families or relatives [19]. It can be inferred from this that perhaps women in rural areas have learnt some level of subjugation to their partners as a means of protecting themselves from IPV.

Concerning age difference between partners, women who were at least 5 years older than their partners and women who were at least 25 years younger than partners were at increased risk of IPV. This is similar to Abramsky’s multi-country study findings which reported a weak association between age gap of at least five years between a woman and her partner and experience of IPV whether the woman or her spouse was older [7]. In contrast, Adebowale’s study of IPV among Nigerian women found that the spousal age difference was not a predictor of IPV although the study reported reduction in the odds of IPV with increasing gap in spousal ages and a spousal age difference of 7.72 ± 4 was associated with experience of IPV [11]. Spousal age differences are believed to affect the perpetration or experience of IPV especially in patriarchal societies where men frequently marry younger women as a means of exerting dominance in marriage [11]. Such age differences particularly when the gap is wide often limits the woman’s negotiating powers in relation to sex and other issues and may predispose to IPV [32, 33]. The status inconsistency theory may also influence the relationship between spousal age difference and IPV particularly for women who are older than their partners. Older women are likely to have access to more resources and better self-agency which may lead to threatened male identity and result in IPV. This may likely be the result of a violent backlash in which the man is at risk of perpetrating violence as a means of taking back control and asserting his position in the relationship [10]. For women who are much younger than their partners, the exertion of dominance by the man [11], the woman’s limited power of negotiation [32, 33] and the possibility of such women having fewer resources than their partners [10, 18] make them less likely or able to exit the relationship and increases their vulnerability to IPV.

### Study limitation

The strengths of our findings may be limited due to unmeasured or residual confounding. However, the methodological strengths behind the findings mitigate against these potential limitations.

## Conclusion

The risk factors for IPV identified in this study were urban residence, living in the north-eastern part of the country, less than secondary educational attainment, partner with less than secondary educational attainment, younger age and being married to a partner at least 5 years younger or at least 25 years older. Further research on the differences in partner’s education attainment and nuances related to region and family relationships are needed for full understanding of these risk factors as well as to ensure deployment of relevant interventions for the prevention of IPV in Namibia.

## Supplementary Information

**Additional file 1**. Full statistical model.

## Data Availability

The dataset used in this study is in the public domain and can be obtained on request from the DHS Program (http://dhsprogram.com/*).*

## Abbreviations

CTS: Conflict tactics scale
DHS: Demographic and health survey
IPV: Intimate partner violence
MCMC: Markov chain Monte Carlo
BIC: Bayesian information criteria
CI: Confidence interval
CI^1^: Credible interval
