# Supplementary material for: Bayesian semi-parametric spatial modelling of intimate partner violence in Namibia using 2013 Demographic Health Survey Data
Source: BMC Womens Health. 2021 Aug 5;21:286. doi: 10.1186/s12905-021-01421-2 (PMC8340378; doi:10.1186/s12905-021-01421-2)
Supplement: Supplementary file 1 — Additional file 1. Full statistical model. [file 12905_2021_1421_MOESM1_ESM.docx]

Appendix 1: Statistical models

## Statistical model formulation

Let $y_{ij}$ be the IPV(IPV) status for a woman $i$ in region $j$. $y_{ij}=1$ if the woman $i$ in region $j$ experienced some form of partner violence and $y_{ij}=0$ otherwise. A vector $X_{ij}=(x_{ij1},x_{ij2},\ldots,x_{ijp})^{'}$ contains $p$ continuous covariate random variables and $Z_{ij}=(z_{ij1},z_{ij2},\ldots,z_{ijr})^{'}$ contains some r categorical variables. In our study, $p=3$ and $r=5$.

This study assumes that the dependent variable, $y_{ij}$ is a Bernoulli distributed random variable with $y_{ij}|p_{ij}\sim Bernoulli(p_{ij})$ with an unknown $E(y_{ij})=p_{ij}$, being related to the covariates through the link function

$$g(p_{ij})=X_{ij}^{'}\beta+Z_{ij}^{'}\theta(1)$$

The link function in this equation is known as the logit link, $\beta$ is the $p$ dimensional vector of coefficients for the continuous random variables, and $\theta$ is an $r$ dimensional vector of coefficients for categorical random variables. In order to assess for both non-linear effects of continuous random variables and spatial autocorrelation in our data we employed a semi-parametric model which utilizes a penalized regression approach(21). The penalized regression approach is a non-parametric method of ordinary least squares (OLS) which relaxes the highly restrictive linear predictor for a versatile semi-parametric predictor(21,25). The flexible semi-parametric predictor is defined by:

$$g(p_{ij})=\sum_{v=1}^{p} f_{v}(x_{ijv})+f_{spat}(s_{j})+Z_{ij}^{'}\theta(2)$$

where $f_{v}(.)$ represents the non-linear twice differentiable smooth function for the continuous covariates and $f_{spat}(s_{j})$ is the variable that denotes the spatial effects for each region. In our study, as in Ngesa et al(21), we consider a convolution approach to the spatial effects. The assumption is that the spatial effects can be decomposed into two pure components, that is, spatially structured and spatially unstructured effects given as $f_{spat}(s_{j})=f_{str}(s_{j})+f_{unstr(s_{j})}$. The final model for our study then becomes:

$$g(p_{ij})=\sum_{v=1}^{p} f_{v}(x_{ijv})+f_{str}(s_{j})+f_{unstr}(s_{j})+Z_{ij}^{'}\theta(3)$$

## Estimation and Prior distributions for unknown parameters

For this study all estimation was done using the full Bayesian approach. In the Bayesian formulation, the specification of the proposed model (model 3) is completed by assigning prior distributions to all the unknown parameters in the model since they are considered random variables. For the fixed covariate parameters, we assume a diffuse prior, that is, $P(\theta)\propto constant$.

For the continuous covariates in the model, these are estimated non-parametrically through smoothness priors. We use the second order Gaussian random walk prior to allow for enough flexibility while penalizing highly oscillating functions as proposed by Lang and Brezger(26). The second order random walk priors can equivalently be written in the form of global smoothness priors as:

$$\beta_{v}|\tau_{v}^{2}\propto exp\left( \frac{-1}{2\tau_{v}^{2}}\beta_{v}^{'}K_{v}\beta_{v} \right) (4)$$

where $K_{v}$ is an appropriate penalty matrix and $\tau_{v}^{2}$ is the variance component for second order random walk $\beta_{v}$. The structure for $K_{v}$ depends on the covariate and smoothness of the function $f_{v}(.)$. In most cases, $ K_{v}$ is rank deficient and hence the prior for $\beta_{v}$ is improper. For the full Bayesian inference as implemented in this study, the unknown variance parameters $\tau_{v}^{2}$ are considered random and are estimated simultaneously with the unknown $\beta_{v}$ (26). The hyper priors are assigned to the variances $\tau_{v}^{2}$ and in this study we assume inverse Gamma priors $IG(a_{v},b_{v})$ with parameters $a_{v}$ and $b_{v}$ (26) chosen in such a way that this prior is weakly informative(24).

For the spatially correlated effects, we assumed the Markov random field prior (mrf). This is one of the simplest (but most frequently used) spatial smoothness prior for the function evaluations $f(s)=\beta_{s}$ and is defined as:

$$f_{str}(s_{i})|f_{str}(s_{j}),i\neq j, \tau_{str}^{2}\sim N\left( \sum_{j\in N_{(i)}} \frac{1}{N_{s}}f_{str}(s_{j}),\frac{\tau_{str}^{2}}{N_{s}} \right) (5)$$

where $N_{s}$ is the number of adjacent regions and $j\in N_{(i)}$ denotes that region $s_{j}$ is in the neighbourhood of region $s_{i}$ (27). The neighborhood can be defined in terms of whether two regions share a border or not. If the two regions share a border then they are neighbors, otherwise they are not. This leads to the intrinsic conditional autoregressive (ICAR) prior distribution(28).

The uncorrelated spatial effects $f_{unstr}(.)$ were assumed to have identically independently distributed (iid) Gaussian random effects, that is, $f_{unstr}(s_{i})|\tau_{unstr}^{2}\sim N\left( 0,\tau_{unstr}^{2} \right)$. For variances $\tau_{str}^{2}$ and $\tau_{unstr}^{2}$, we assume inverse Gamma priors. Formally, the priors for $f_{str}$ and $f_{unstr}$ can be incorporated into equation (4).

## Posterior distribution

Posterior distribution is the distribution of the parameters given the data. Fully Bayesian inference is based on the analysis of posterior distribution of the model parameters. Full Bayesian inference derives its estimates by sampling from this posterior distribution. Generally, the posterior distribution is usually high dimensional and analytically intractable. This problem is solved through the use of MCMC simulation techniques, whereby samples are drawn from the full conditional of parameters given the data. Under conditional independence assumptions, the posterior distribution for the Bernoulli model is given by Bayes’ Theorem:

$$P(\beta,\tau^{2},\theta|data)\propto L(data|\beta,\tau^{2},\theta)P(\beta,\tau^{2},\theta)$$

$$=L(data|\beta,\tau^{2},\theta)\times\left\{ \prod_{v=1}^{p} p(\beta_{v}|\tau_{v}^{2})p(\tau_{v}^{2}) \right\}p(\theta)$$

where the quantity $p(\beta,\tau^{2},\theta)$ is the prior density function and $L(data|\beta,\tau^{2},\theta)$ is the likelihood for the Bernoulli distribution. Thus, the full posterior distribution for our Bayesian estimation is given by

$$p(\beta,\tau^{2},\theta)\propto\prod_{i=1}^{n} p_{ij}^{y_{ij}}(1-p_{ij})^{1-y_{ij}}\times exp\left\{ \frac{-1}{2\tau_{v}^{2}}\beta_{v}^{'}K\beta_{v} \right\}\times\prod_{v=1}^{p} \frac{1}{\Gamma(a_{v})b_{v}^{a_{v}}}(\tau_{v}^{2})^{-(a_{v}+1)}exp\left( \frac{-b_{v}}{\tau_{v}^{2}} \right)\times p(\theta)$$

For updating the full conditionals of parameters, we used the MCMC sampling scheme of the iteratively weighted least squares (IWLS) proposals developed by Gameman for generalized linear mixed models together with the Metropolis-Hastings algorithm. All the analysis in this study were carried out using R version 3.5.2 implementing the R2BayesX package. For the MCMC procedure, 12 000 iterations were used with the initial 1000 discarded for burn-in and thereafter keeping every tenth sample value. We assessed the MCMC convergence of the model parameters using trace plots and autocorrelation (ACF) plots.

For our purposes we fit a single model to assess the non-linear effects of woman’s age, woman’s years of education and age difference with partner on experiencing some form of intimate partner violence. We also use the woman’s region in the model to assess the unobserved spatial effects as well as uncorrelated spatial heterogeneity effects on the outcome. Fixed effects like education level and place of residence are also assessed.
